# The Influence of Children’s Discrimination Experiences on Parents’ Mental and Self-Rated Health: Results from the National Health Interview Survey

**DOI:** 10.3390/children11070830

**Published:** 2024-07-08

**Authors:** Elizabeth Jelsma, Nema Kebbeh, Mahnoor Ahmad

**Affiliations:** Psychological, Health, & Learning Sciences, University of Houston, 3657 Cullen Blvd, Room 491, Houston, TX 77204, USA

**Keywords:** race/ethnicity, sexual orientation/gender identity, vicarious discrimination, parents, mental health, physical health

## Abstract

Background: This study assessed the associations between children’s experiences of discrimination based on race/ethnicity and sexual orientation/gender identity and their parents’ anxiety, depressive symptoms, and self-rated health. Methods: Our sample included 3910 parents with at least one child between 12 and 17 years of age. Data were pooled from the 2021 and 2022 waves of the National Health Interview Survey. Path analysis with maximum likelihood (ML) estimation and bootstrapping were used to examine the indirect pathways from child-experienced discrimination to parental self-rated health through parental anxiety and depressive symptoms. Results: Child-experienced discrimination based on race/ethnicity was associated with worse parental self-rated health via pathways through higher parental anxiety (*p* = 0.003) and depressive symptoms (*p* < 0.001). Child-experienced discrimination based on sexual orientation/gender identity was also associated with worse parental self-rated health via pathways through higher parental anxiety (*p* = 0.002) and depressive symptoms (*p* = 0.001). Conclusions: The results show a strong association between children’s experiences of discrimination and parental self-rated health through pathways of poor parental mental health. Findings demonstrate the need to reduce discrimination directed at children and to facilitate mental health support for parents of children experiencing discrimination to improve family well-being.

## 1. Introduction

Vicarious discrimination is an indirect, distressing experience that occurs when an individual learns about or witnesses discrimination that occurs among their loved ones or strangers [1]. The Linked Lives principle of the life course framework [2] suggests that when one person experiences hardship, it is likely to cause distress among those who share social bonds with them. Indeed, previous research shows that children are negatively affected by their parent experiencing discrimination [3,4], as well as the opposite [5,6]. While direct links have been found between child-experienced discrimination and parental outcomes, the current literature lacks (1) consideration of vicarious discrimination based on other marginalized identities beyond race/ethnicity, (2) substantial samples of fathers, and (3) elucidation of the mechanisms by which child-experienced discrimination might influence parental health. The current study attempts to address these gaps by accounting for child-experienced discrimination based on race/ethnicity and sexual orientation/gender identity, utilizing a nationally representative sample of U.S. parents that is 40% fathers, and examining indirect pathways by which psychological distress from vicarious discrimination might explain its influence on parental physical health. As parental mental and physical distress also pose a risk for adverse effects on child health [7,8], it is necessary to identify the upstream predictors which might explain parental mental and physical distress in order to optimize health within the entire family.

Adolescence is a critical period in sexual orientation and gender identity development [9]. Recent trends show that lesbian, gay, bisexual, or transgender (LGBT) individuals are coming out at younger ages compared to previous decades, with approximately 9.5% of youth ages 13–17 in the United States identifying as LGBT [10]. This increased visibility, however, also comes with a higher risk of discrimination. A survey conducted by The Trevor Project [11] revealed that a staggering 73% of LGBT youth reported experiencing discrimination based on their sexual orientation or gender identity at least once in their lifetime. Despite being a prevalent stressor for a large population of youth in the United States, little research has investigated the influence of this stressor on the family of LGBT youth. Previous research examining parents’ vicarious exposure to their children’s experiences of racial/ethnic discrimination shows increased parental depressive symptoms and worse self-rated health [5,12]. Child-experienced discrimination based on sexual orientation and gender identity is also a distressing form of discrimination for children, and nurturing parents who generally desire to prevent harm coming to their children will also presumably be pained and negatively impacted by discriminatory treatment against their LGBT child.

In their review of discrimination and health from a life-course perspective, Gee and colleagues [13] suggest that “for parents, discrimination against their child may be more stressful than discrimination against the parents themselves”. Vicarious discrimination, or discrimination experienced indirectly by hearing about or observing it happen to someone else, has clear negative implications for physical health [14,15]. According to the “linked lives” concept of life-course theory [13,16], individual lives are lived interdependently. Thus, occurrences that affect one person also affect others in their network. Parents may experience unease, shame, grief, exhaustion, bitterness, and/or rage in response to their child being treated unfairly upon realizing they cannot protect them from all possible bias-based harm [17,18]. Psychological distress may offer one explanation of how vicarious stressors get under the skin and perpetuate negative health consequences, particularly for parents who are grappling with the reality of their child being the target of discrimination. Mental and physical health are inextricably linked, with advances or declines in one leading to the same in the other [19]. For example, psychological distress can speed up biological aging [20] or encourage poor nutritional habits [21], therefore perpetuating higher rates of disease. Poor physical health is also associated with a lower perceived need for mental health services due to the belief that mental health and physical health are not linked [22], despite physical symptoms often being the result of chronic poor mental health [23]. Given the established mediating role of psychological distress in the relationship between interpersonal stress and poor physical health [24], more research is needed to investigate the mediating role of psychological distress when the stress is experienced indirectly or vicariously. As parent and caregiver health are essential to children’s healthy development [25], improving parental mental and physical health will also have positive downstream consequences for their children.

### The Current Study

From a “linked lives” perspective [2,16], experiences of discrimination faced by one person may vicariously affect their loved ones [13]. Parents, who generally strive to protect their children from harm, may be particularly distressed by their children’s experiences of discrimination. While there is evidence of this phenomenon with vicariously experienced racial/ethnic discrimination, there lacks any research documenting the ripple effects of discrimination based on sexual orientation or gender identity across family systems. In addition, fathers have been grossly underrepresented in research examining vicarious discrimination within families [3,4,5,26]. To address these important gaps in the research, the current study uses path analysis in a structural equation modeling framework to test how parental anxiety and depressive symptoms may mediate the influence of two forms of child-experienced discrimination (based on race/ethnicity and sexual orientation/gender identity) on parental self-rated physical health among a nationally representative sample of parents of 12–17-year-olds (40% fathers). First, we hypothesized that child-experienced discrimination based on race/ethnicity and sexual orientation/gender identity would predict worse parental self-rated physical health (Hypothesis 1). Second, we hypothesized that parental anxiety and depression would mediate the associations between both forms of discrimination and parental self-rated physical health (Hypothesis 2).

## 2. Materials and Methods

### 2.1. Data

We used data from the 2021 and 2022 waves of the National Health Interview Survey (NHIS) due to the inclusion of child-experienced discrimination in these waves. Data are collected annually by The National Center for Health Statistics (NCHS) of the Centers for Disease Control and Prevention (CDC). We used the Public Use version of the NHIS, which is publicly available to download from the Integrated Public Use Microdata Series (IPUMS) Health Surveys. The NHIS data are comprised of a “Sample Child file” that contains data on children aged 17 and under (reported by an adult), and a “Sample Adult file” that contains data on individuals aged 18 and over. Between 2021 and 2022, 11,278 sample adults completed one survey for themselves and a separate survey for one of their children. Thus, adult and child responses were matched via unique household identifiers. If the sample adult was not the sample child’s parent, that household was excluded. Only interviews for sample children aged 12–17 assessed discrimination because of sexual orientation or gender identity (*N* = 3910). Although interviews for all children assessed discrimination based on race or ethnic group, the current analysis only included households with children aged 12–17 to compare the two forms of child-experienced discrimination for parental mental and physical health. Therefore, the sample size for the current study is *N* = 3910.

### 2.2. Measures

In the “Sample Child” survey, under the “Stressful Life Events (SLE)” module, adult respondents were asked if the respondent thought “Has anyone ever treated or judged (sample child name) unfairly because of his/her race or ethnic group?” and “Has anyone ever treated or judged (sample child name) unfairly because of his/her sexual orientation or gender identity?” Response options were coded as 0 = “no” and 1 = “yes”. These measures of child discrimination have been used in several previous studies [27,28,29]. In the “Sample Adult” survey, respondents were asked “How often do you feel worried, nervous, or anxious? Would you say daily, weekly, monthly, a few times a year, or never?” They were also asked “How often do you feel depressed?” with the same response options. Responses were coded as 0 = “never”, 1 = “a few times a year”, 2 = “monthly”, 3 = “weekly”, and 4 = “daily” for each question separately. Parents were then asked “Would you say your health in general is excellent, very good, good, fair, or poor?” Higher responses were recoded to reflect worse health. Covariates from the “Sample Child” survey include child age (12–17 years), sex (0 = male and 1 = female), and race (Hispanic, Non-Hispanic White, Non-Hispanic Black/African American, Non-Hispanic Native American/Alaka Native only, or Non-Hispanic Other single or multiple races). Covariates from the “Sample Adult” survey include parent race, age (18–65 years), sex (0 = male and 1 = female), marital status (0 = not married and 1 = married), educational attainment (higher scores reflecting higher educational attainment), and employment status (0 = unemployed and 1 = employed).

### 2.3. Statistical Analysis

**Figure 1:**
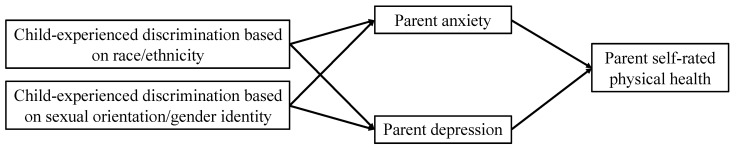
Conceptual mediation model linking both forms of child-experienced discrimination, parent mental health, and parent self-rated physical health.

Variables were prepared in Stata/MP 18 [30], and path analyses were conducted in Mplus 8.10 [31] using maximum likelihood estimation with bootstrapped standard errors based on 5000 samples. Missing data were handled with full information maximum likelihood (FIML) estimation, enabling us to include all available data [32]. Maximum likelihood estimation with bootstrapped standard errors was used due to non-normality in the categorical independent variables (child-experienced discrimination based on race/ethnicity and sexual orientation/gender identity) and the tests of indirect effects [33,34]. When numerical integration is required, chi-square and related fit statistics are not available. Notably, the mediators and distal outcomes were continuous. All estimates were obtained using the complex survey weights (provided by NHIS) for each sample adult, as most of the variables in the estimated models come from the sample adult interviews. Sampling weights from NHIS assigned to sample children were not used in these analyses, as only one “weight” value could be used per household. To test whether both forms of child-experienced discrimination were related to parent self-rated health indirectly through parental anxiety and depression, we analyzed the path model shown in Figure 1.

## 3. Results

### 3.1. Descriptive Results

Almost 8% of parents (*N* = 298) reported that their child had experienced racial/ethnic discrimination, and 3% (*N* = 111) reported that their child had experienced discrimination based on sexual orientation or gender identity. Ten percent of the sample of 12–17-year-olds (*N* = 376) experienced some form of discrimination (9% experienced one form, and 1% experienced both forms). The mean child age was 14.58 years (SD = 1.69 and range 12–17 years) and mean parent age was 45.55 years (SD = 6.94 and range 18–65 years). Table 1 presents the descriptive statistics of the study variables for the total analytic sample and by children who have and have not experienced discrimination.

### 3.2. Direct and Indirect Links among Central Study Constructs

When testing the direct effects of child-experienced discrimination based on race/ethnicity and sexual orientation/gender identity for parent self-rated physical health (Hypothesis 1), both forms of discrimination were related to worse self-rated physical health (*b* = 0.182, *p* = 0.008 and *b* = 0.242, *p* = 0.025, respectively). When examining parental anxiety and depression as mechanisms linking both forms of child-experienced discrimination and parental self-rated physical health (Hypothesis 2), significant indirect effects were found. Table 2 summarizes the indirect paths from both forms of child-experienced discrimination to parental self-rated physical health through parental anxiety and depression. Specifically, child-experienced discrimination based on race/ethnicity had significant indirect effects on parental self-reported physical health through parental anxiety (*p* = 0.003) and parental depression (*p* < 0.001), and the magnitude of the indirect effects corresponded to 54% of the total effects of child-experienced discrimination based on race/ethnicity on parental self-rated physical health. Next, there were significant indirect effects of child-experienced discrimination based on sexual orientation/gender identity to parental self-reported physical health through parental anxiety (*p* = 0.002) and parental depression (*p* = 0.001), and the magnitude of the indirect effects corresponded to 62% of the total effects of child-experienced discrimination based on sexual orientation/gender identity on parental self-rated physical health.

## 4. Discussion

To the best of our knowledge, this is the first study to examine the influences of child-experienced discrimination based on race/ethnicity and sexual orientation/gender identity on both parental mental and physical health. Our findings indicate that both forms of child-experienced discrimination are distressing for parents, and this mental distress may explain the resulting physical distress. Our findings are in concordance with other studies suggesting a “linked lives” perspective, finding that discrimination directed toward others can also be distressing for their loved ones [5,12,13]. Our study furthers existing research in several ways. First, we extend the research on child-experienced discrimination for parental well-being by including discrimination based on sexual orientation/gender identity, in addition to discrimination based on race/ethnicity. Second, we utilized a nationally representative sample of parents, 40% of which were fathers, bolstering the representation of fathers in family-centered research. Third, we examined underlying mechanisms of how the previously established relationship between child-experienced discrimination and parental self-rated physical health [5] might in fact be explained by parental anxiety and depression, thus identifying important and more accurate points of possible prevention and intervention.

Research shows that discrimination significantly affects families, and parents find it particularly distressing when they cannot help their children manage such experiences [35]. Parents often experience a range of emotions—disillusionment, sadness, anxiety, anger, and frustration—when their children face discrimination and when they are unable to secure mental health support for them [35]. For parents who have themselves faced discrimination across various aspects of their identity, it is especially hard to see their children exhibit face marginalization and discrimination, as this situation may remind them of their own feelings of powerlessness and trigger emotional distress [36]. And parents who themselves have not experienced discrimination might lack the resiliency and coping abilities needed to psychologically deal with learning about their children’s marginalizing experiences [37].

Discrimination based on sexual orientation or gender identity is a significant stressor for parents of adolescents, especially as more adolescents identify as LGBTQ. The Centers for Disease Control and Prevention reports that the number of high school students identifying as LGBT has increased from 11% in 2015 to 26% in 2021 [38]. This increase means more parents are likely to be confronted with their LGBTQ children’s experiences of discrimination. Seeing their children suffer from discrimination due to being LGBTQ can lead to increased depressive symptoms in parents, exacerbated by feelings of stress and helplessness in protecting their children [39]. Parents can also experience high levels of psychological distress from these vicarious experiences, indicating a significant emotional burden that encompasses both their own and their children’s experiences [15]. This stress is often heightened by feelings of overload and depressive symptoms, underscoring the extensive negative impacts of vicarious discrimination [40].

Our findings suggest that psychological distress in the form of depressive symptoms and anxiety may explain the influence of child-experienced discrimination on parental self-rated physical health. This is an important mechanism to understand, as early detection of poor mental health and subsequent support seeking can minimize symptoms and even prevent the internalization of symptoms and manifestation into physical symptoms. Somatic symptoms very often co-occur with psychological distress and often need treatment beyond routine mental health treatment [41]. Early detection is key to prevention and management of somatic symptoms that often cannot be explained by other physical conditions. The quality of life of an individual greatly improves when symptoms do not go unnoticed and can be treated at the root. Treating mental health symptoms early will also help prevent somatic symptoms from appearing or progressing.

## 5. Conclusions

Our results should be understood within some limitations. First, due to cross-sectional data, we were not able to detect whether parents’ depression and anxiety preceded physical distress, and vice versa. Therefore, we cannot suppose causation. Second, we were limited by each form of discrimination, parental anxiety, depression, and self-rated physical health all being measured with only one item. Relatedly, future research should also incorporate other forms of interpersonal discrimination experienced by children (e.g., based on immigration status, language, religion, and disability) as well as systemic racism and discrimination to understand their vicarious influence on parents. However, our analyses using nationally representative data with almost equal proportions of mothers and fathers still demonstrate robust links between child-experienced discrimination and parental mental and physical health.

This research offers significant insight into the links among child-experienced discrimination and parental mental and physical health. Our results indicate that parents of children who experience discrimination based on race/ethnicity and sexual orientation/gender identity may have noteworthy mental and physical health difficulties. While more longitudinal studies are necessary to clarify how child-experienced discrimination influences parental mental and physical distress, our results indicate that parents of children who experience discrimination should be identified for mental health interventions to avoid downstream adverse physical health consequences. The results of this study do support that discrimination experienced by one individual may indirectly affect others in their network. Experiences of stress and marginalization ripple across family systems in a bidirectional way and disrupt healthy functioning. Children distressed by discrimination may cause parents to worry and experience distress, and having anxious and depressed parents will also increase risk of adverse child outcomes. Reducing discrimination based on all marginalized identities, and promoting practices and policies which promote belonging for all children, have the potential to improve and promote the well-being of not just the children, but of entire families.

## Figures and Tables

**Table 1:** Descriptive statistics.

	Percentage of Sample		
	All (*N* = 3910)	Child Did Not Experience Discrimination (*N* = 3442)	Child Did Experience Discrimination (*N* = 376)
Parent’s self-reported anxiety			
Never	26.2	27.7	14.5
A few times a year	32.3	32.9	30.0
Monthly	13.2	13.0	15.5
Weekly	15.1	14.8	16.6
Daily	12.6	11.6	23.3
Parent’s self-reported depression			
Never	54.9	57.0	39.1
A few times a year	29.2	28.8	34.3
Monthly	7.5	6.9	13.4
Weekly	4.9	4.6	7.5
Daily	3.0	2.7	5.6
Parent’s self-reported physical health			
Poor	1.7	1.7	1.6
Fair	8.7	8.1	13.6
Good	27.6	27.1	30.9
Very good	38.3	38.5	36.2
Excellent	23.8	24.5	17.8
Parent’s sex			
Male	39.0	39.3	65.2
Female	61.0	60.7	34.8
Parent’s race			
Hispanic	21.2	21.5	18.6
Non-Hispanic White	55.1	56.1	48.4
Non-Hispanic Black/African American	9.6	8.5	19.9
Non-Hispanic American Indian/Alaska Native only	0.6	0.6	0.8
Non-Hispanic Other single and multiple races	2.3	1.9	4.8
Marital status			
Married	69.9	71.2	58.5
Not married	29.8	28.6	41.5
Employment status			
Employed	82.2	82.0	84.0
Not employed	17.6	17.8	16.0
Parent’s educational attainment			
Did not graduate high school	10.1	10.2	9.9
High school graduate or GED	19.5	20.0	17.6
Some college, no degree	14.0	13.9	13.9
Associate degree	11.8	3.8	12.8
Bachelor’s degree	26.3	26.6	24.1
Master’s degree	14.1	13.8	16.3
Professional school or doctoral degree	4.1	3.8	5.3
Child’s sex			
Male	51.2	52.0	44.7
Female	48.7	48.0	55.1
Child’s race			
Hispanic	23.4	23.4	22.3
Non-Hispanic White	50.7	52.9	34.3
Non-Hispanic Black/African American	9.5	8.2	75.8
Non-Hispanic American Indian/Alaska Native only	0.6	0.6	1.3
Non-Hispanic Other single and multiple races	5.2	4.3	12.5

**Table 2:** Path model of indirect effects from child-experienced discrimination to parent self-rated health.

Paths	Est.	SE	95% CI	*p*-Value
Child-Experienced Discrimination Based on Race/Ethnicity to Parent Self-Rated Health				
** Child discrimination to Parental Anxiety to Parent Health**	**0.028**	**0.010**	**0.012, 0.051**	**0.003**
** Child discrimination to Parental Depression to Parent Health**	**0.071**	**0.020**	**0.034, 0.113**	**0.000**
Total indirect effects	0.099	0.025	0.050, 0.151	0.000
Total direct effects	0.084	0.067	−0.044, 0.219	0.212
Total effects	0.183	0.068	0.048, 0.317	0.008
Child-Experienced Discrimination Based on Sexual Orientation/Gender Identity to Parent Self-Rated Health				
** Child discrimination to Parental Anxiety to Parent Health**	**0.048**	**0.016**	**0.023, 0.084**	**0.002**
** Child discrimination to Parental Depression to Parent Health**	**0.103**	**0.032**	**0.046, 0.172**	**0.001**
Total indirect effects	0.151	0.040	0.078, 0.236	0.000
Total direct effects	0.092	0.097	−0.099, 0.285	0.342
Total effects	0.242	0.109	0.025, 0.454	0.027

Note: Unstandardized path parameters are presented, and significant indirect pathways are bolded. Bootstrapped confidence intervals are based on 5000 samples.

## Data Availability

We used the Public Use version of the NHIS, which is publicly available to download from IPUMS Health Surveys by accessing the following URL: https://healthsurveys.ipums.org/, accessed on 1 November 2023.
